# Supplementary material for: Chromosomally Unstable Gastric Cancers Overexpressing Claudin-6 Disclose Cross-Talk between HNF1A and HNF4A, and Upregulated Cholesterol Metabolism
Source: Int J Mol Sci. 2022 Nov 12;23(22):13977. doi: 10.3390/ijms232213977 (PMC9694805; doi:10.3390/ijms232213977)
Supplement: Supplementary file 1 [file ijms-23-13977-s001.zip › ijms-2000596-supplementary.pdf]

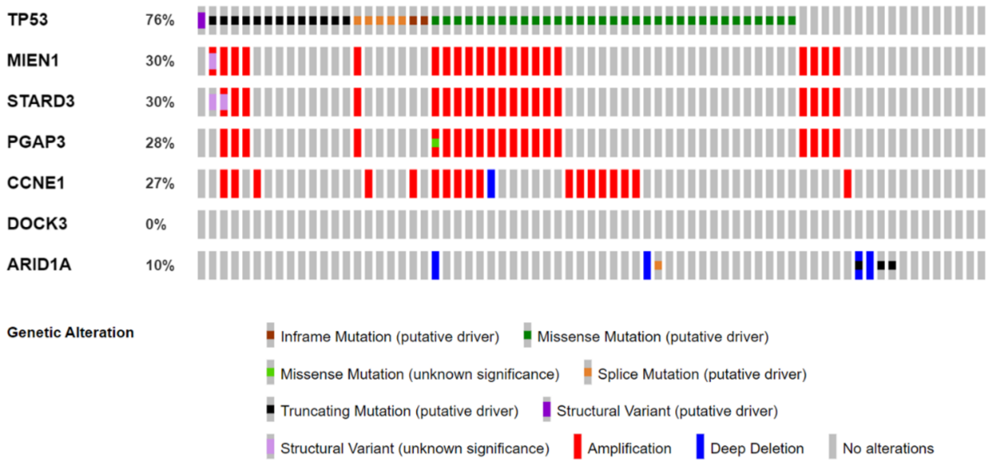

Figure S1- Oncoprint of genomic copy number alterations and mutations in Cldn-6^{high} gastric tumors.

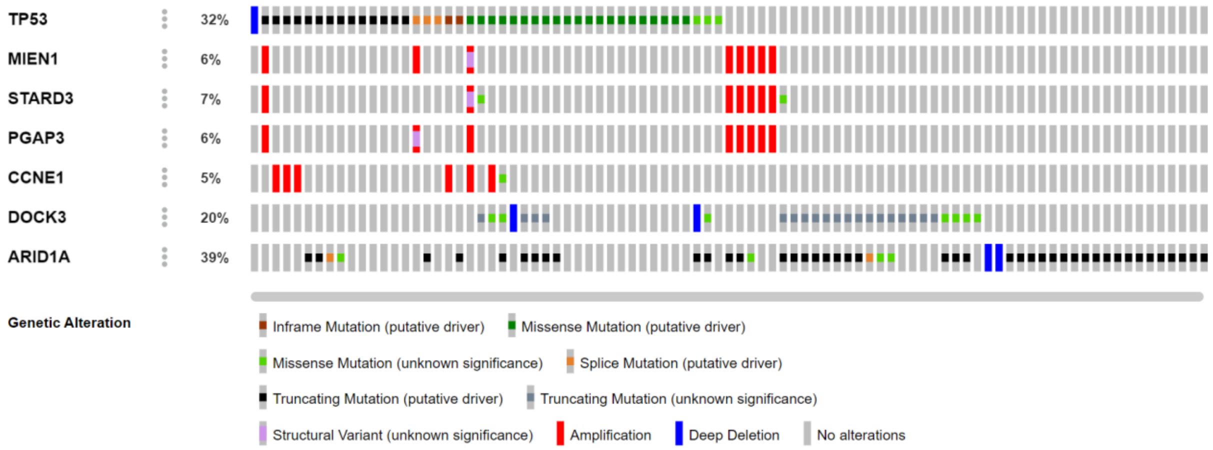

Figure S2 - Oncoprint of genomic copy number alterations and mutations in Cldn-6^{low} gastric tumors.
